# P-1325. A Comparative Analysis of Ceftazidime-avibactam and Meropenem-vaborbactam in the Treatment of Carbapenem-resistant Enterobacterales Infections

**DOI:** 10.1093/ofid/ofaf695.1513

**Published:** 2026-01-11

**Authors:** Kyle G Crooker, Alana Pinheiro Alves, Denise Araujo, Johnny Zakhour, Madison Patrus, Anita Shallal, Geehan Suleyman, Ulyana Malovana

**Affiliations:** Henry Ford Hospital, Detroit, MI; Henry Ford Health, Farmington Hills, MI; Henry Ford Health, Farmington Hills, MI; Henry Ford Health, Farmington Hills, MI; Henry Ford Health, Farmington Hills, MI; Henry Ford Hospital, Detroit, MI; Henry Ford Health, Farmington Hills, MI; Henry Ford Hospital, Detroit, MI

## Abstract

**Background:**

Novel β-lactam/β-lactamase inhibitor (BL/BLI) combinations, including ceftazidime-avibactam (CZA) and meropenem-vaborbactam (MVB), are the treatment of choice for carbapenem-resistant organisms (CRO). However, comparative data remain scarce. This study aims to compare outcomes among patients with confirmed CRO infections treated with CZA and MVB.

**Table 1:**
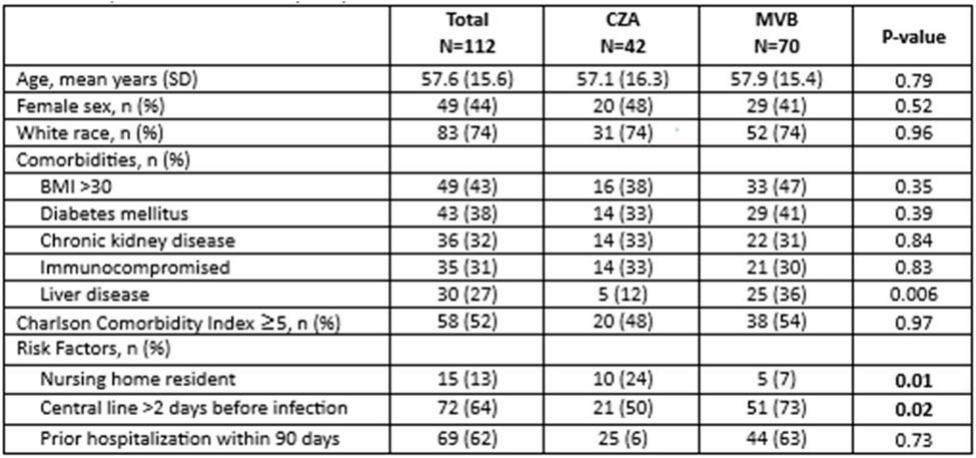
Demographic data and risk factors comparing patients receiving ceftazidime-avibactam (CZA) versus meropenem-vaborbactam (MVB)

**Table 2:**
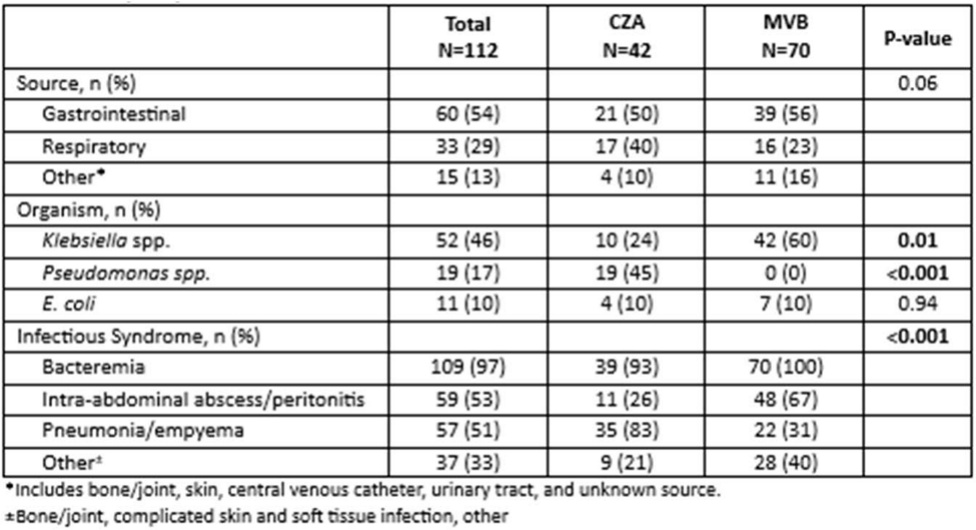
Infection characteristics of patients receiving ceftazidime-avibactam (CZA) versus meropenem-vaborbactam(MVB)

**Methods:**

Retrospective cohort study of inpatients treated with CZA or MVB from January 2018 to December 2024 at Henry Ford Health in Southeast Michigan. Hospitalized adults receiving >3 days of CZA or MVB for index culture-confirmed CRO infection were included. Readmitted patients receiving treatment for prior CRO infection were excluded. Primary outcome was all-cause 30-day mortality; secondary outcomes included length of stay (LOS), 30-day relapse, microbiologic cure, development of resistance after treatment, and antibiotic adverse events. Microbiologic cure was defined as no repeat CRO growth on later cultures.

**Table 3:**
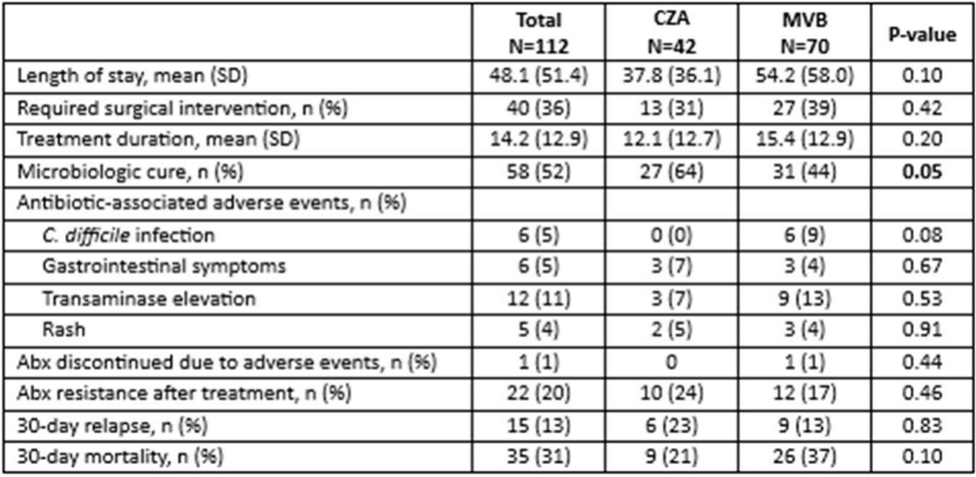
Clinical outcomes of patients receiving ceftazidime-avibactam versus meropenem-vaborbactam

**Results:**

Among 350 patients evaluated, 112 (32%) met inclusion criteria, 42 (38.5%) received CZA and 70 (62.5%) MVB (Table 1). Most were male and white with mean age of 57 years. Most patients had a high Charlson Comorbidity Index; liver disease was more prevalent in the MVB group. The gastrointestinal tract was the most common source, with Klebsiella spp as the most common pathogen among both groups (Table 2). Nearly all patients had bacteremia; lower respiratory tract infection was more frequent in the CZA group, while intra-abdominal infection was more prevalent in the MVB group. Primary and secondary outcomes were similar between the two groups, except for microbiologic cure, which was higher in the CZA group (66% vs. 44%, p=0.05) (Table 3). Approximately one-third of patients required surgical intervention. Treatment duration was similar between groups. Both had prolonged LOS and high mortality, with a higher rate in the MVB group, though the difference was not statistically significant.

**Conclusion:**

CZA and MVB were overall equivalent in terms of CRO infection characteristics, but patients receiving CZA had greater rates of microbiologic cure and a trend toward lower rates of C difficile and 30-day mortality.

**Disclosures:**

All Authors: No reported disclosures

